# Stereotactic body radiation therapy for hepatocellular carcinoma: Practice patterns, dose selection and factors impacting survival

**DOI:** 10.1002/cam4.1948

**Published:** 2019-01-31

**Authors:** Jared R. Robbins, Ryan K. Schmid, Abdulrahman Y. Hammad, Thomas Clark Gamblin, Beth A. Erickson

**Affiliations:** ^1^ Department of Radiation Oncology University of Arizona College of Medicine Tucson Arizona; ^2^ Department of Radiation Oncology Medical College of Wisconsin Milwaukee Wisconsin; ^3^ Division of Surgical Oncology Department of Surgery Medical College of Wisconsin Milwaukee Wisconsin

**Keywords:** hepatocellular carcinoma, practice patterns, radiation, stereotactic body radiation therapy

## Abstract

**Background:**

Stereotactic body radiation therapy (SBRT) is an emerging option for unresectable hepatocellular carcinoma (HCC) without consensus regarding optimal dose schemas. This analysis identifies practice patterns and factors that influence dose selection and overall survival, with particular emphasis on dose and tumor size.

**Materials/Methods:**

Query of the National Cancer Database (NCDB) identified patients with unresectable, nonmetastatic HCC who received SBRT from 2004 to 2013. Biological Effective Dose (BED) was calculated for each patient in order to uniformly analyze different fractionation regimens.

**Results:**

A total of 456 patients met the inclusion criteria. The median BED was 100 Gy (22.5‐208.0), which corresponded to the most common dose fractionation (50 Gy in five fractions). Various factors influenced dose selection including tumor size (*P* < 0.001), tumor stage (*P* = 0.002), and facility case volume (<0.001). On multivariate analysis, low BED (<75 Gy, HR 2.537, *P* < 0.001; 75‐100 Gy, HR 1.986, *P* = 0.007), increasing tumor size (HR 1.067, *P* = 0.032), elevated AFP (HR 1.585, *P* = 0.019), stage 3 (HR 1.962, *P* < 0.001), low‐volume facilities (1‐5 cases HR 1.687, *P* = 0.006), and a longer time interval from diagnosis to SBRT (>2 to ≤4 months, HR 1.456, *P* = 0.048; >4 months, HR 2.192, *P* < 0.001) were associated with worse survival.

**Conclusion:**

SBRT use is increasing for HCC, and multiple regimens are clinically employed. Although high BED was associated with improved outcomes, multiple factors contributed to the dose selection with favorable patients receiving higher doses. Continued efforts to enhance radiation planning and delivery may help improve utilization, safety, and efficacy.

## 1 INTRODUCTION

Hepatocellular carcinoma (HCC) is a primary liver malignancy often arising from etiologies that cause chronic inflammation and cirrhosis. Its relatively poor prognosis is reflected by its rank as third in cancer mortality worldwide.^1^ HCC is a growing problem with tripling incidence in the United States since 1980.^2^ Various options are available for treatment, with transplantation or partial hepatectomy being the standard of care. Unfortunately, less than 30% of people are candidates for surgical therapies due to disease extent, location of tumor, and other medical comorbidities.^2^, ^3^ For the majority with inoperable HCC, therapeutic options include radiofrequency ablation (RFA), percutaneous ethanol injection, and arterial embolization treatments: bland, conventional chemoembolization, or radioembolization. These modalities can be limited by various factors including invisibility of tumor on ultrasound, coagulopathy, tumor size, and proximity to structures such as the gallbladder, hepatic hilum, or vasculature.^4^

Radiation therapy has not traditionally been considered as a viable option for the treatment of HCC due to the sensitivity of the liver to radiation; however, advances in radiation technique (3D‐conformal, intensity‐modulated radiation therapy) and delivery (respiratory motion management, image guidance) have resulted in the development of stereotactic body radiation therapy (SBRT).^5^ SBRT delivers high‐dose ablative radiation to precisely delineated targets with tight conformity and a steep dose falloff in a limited number of treatment sessions. The sparing of the normal liver and other surrounding structures from high doses while delivering significant dose to the tumor results in a safe and effective therapy with limited toxicity. As this technique has evolved, several small prospective studies and single institutional reviews have been published.^6^, ^7^, ^8^, ^9^, ^10^, ^11^, ^12^, ^13^, ^14^, ^15^, ^16^ These results show promise in terms of local control and overall survival, especially considering the limited treatment options available for most of the patients receiving SBRT.

Despite the success and increased application of SBRT techniques for treating unresectable HCC, there remain important questions about the optimal radiation schema, patient selection, and factors predictive of survival outcome. To help address these questions, we have used the National Cancer Database (NCDB) to analyze the largest cohort of HCC patients receiving SBRT therapy.

## 2 METHODS

This Institutional Review Board (IRB)‐approved study surveyed the National Cancer Database (NCDB) to identify HCC patients through utilization of the International Classification of Diseases‐Third revision (ICD‐3) histology codes, 8170‐8175 (HCC) combined with site‐specific code, C22.0 (liver). This database, a result of a joint program between the American College of Surgeon (ACS)‐Commission on Cancer (COC) and the American Cancer Society, captures a wide variety of clinicopathologic characteristics from registries of more than 1500 CoC‐accredited hospitals in the United States and includes approximately 65% of all new liver malignancies.

The final cohort included patients with nonmetastatic HCC, defined as, stage I/II/III disease according to the American Joint Committee on Cancer (AJCC) staging system (7th edition), who received SBRT, as defined by delivery of ≤five fractions of radiation with fractional doses higher than standard palliative regimens consistent with current practices and trends due to insurance reimbursement in the United States. The clinicopathologic variables extracted included patient demographics, Alpha‐Fetoprotein level (AFP), Charlson Comorbidity Index (CCI), tumor size, disease stage, radiation dose, and number of treatments. Biological Effective Dose (BED) was calculated with the following formula^17^:

$$\text{BED}=\text{n}d\left[1+\frac{d}{\alpha /\beta }\right]$$

With n = number of treatment, *d* = dose per fraction, and *α*/*β* ratio of 10 for each case to account for the various fractionation schemes that was used.

Facility case volume was calculated by counting the number of cases associated with each facility ID over the study period and classified as high (>20 cases), moderate (6‐20 cases), and low (five or fewer cases). The NCDB records the receipt of chemotherapy, but does not give detailed information about the specific types or delivery methods, so some patients coded as receiving chemotherapy may have actually received transarterial chemoembolization (TACE), which is more common in this population.

Descriptive analysis of the frequency of different fractionation schemas, BED frequencies, and associations with tumor size and survival were performed. Univariate comparisons were made using chi‐square, ANOVA, and/or t tests. While all 456 patient data sets were used to characterize the cohort, only the 355 patients with follow‐up and status at last known contact were included in the survival analysis. Kaplan‐Meier curves and log‐rank tests were used to examine survival outcomes, while multivariate Cox proportional hazards model was used to identify predictors of survival. Overall Survival (OS) was calculated from the time of SBRT until the time of death. Hazards ratio (HR) and 95% confidence intervals (CI) were reported for the Cox regression analysis. Alpha was established at 0.05 for all tests and *P* < 0.05 was considered significant. Statistical analyses were performed using SPSS version 24.0 (IBM Corp, New York, NY).

## 3 RESULTS

**Figure 1:**
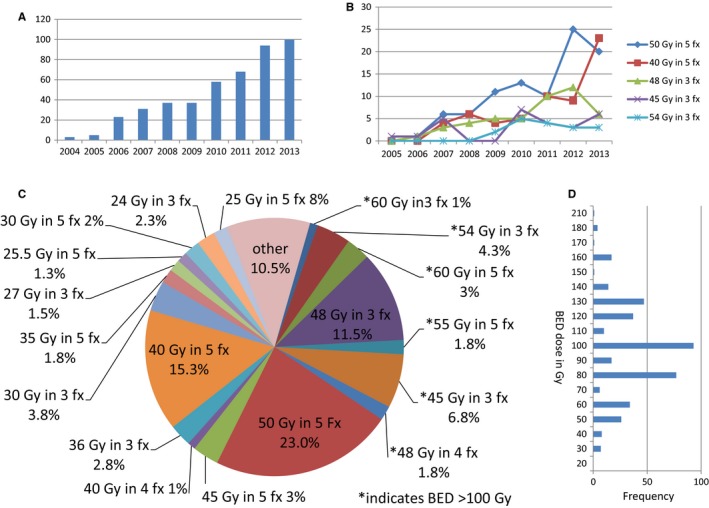
A, Incidence of SBRT use from 2004 to 2013; B, Use of common fractionation regimens over the study period; C, Distribution of SBRT dose regimens; D, Distribution of BED

A total of 456 patients were found to have received liver SBRT for HCC. The median patient age and tumor size were 62 years (27‐90) and 3.2 cm (0.6‐17), respectively. Table 1 shows patient demographics for the whole group as well as divided into groups by BED dose. Most cases were primary lesions, but 21 patient (<5%) had surgical procedure prior to SBRT (partial liver resection in 10 patients and ablation in 11 patients; median time from procedure to SBRT of 93 days) and 132 patients (29%) received some form of chemotherapy prior to SBRT (median time from chemotherapy to SBRT was 73 day). Patients with prior therapy had a longer time from diagnosis to SBRT (prior surgery: median time of 151 days vs 80 days for no prior surgery *P* = 0.02951; prior chemotherapy: median 131 days vs 72 days for no prior chemotherapy *P* < 0.001). During the study period from 2004 to 2013, the use of SBRT for liver HCC increased dramatically from four cases in 2004 to 100 cases in 2013. Figure 1 shows the use of SBRT over the study period, the distribution of each dose‐fractionation schema, and the distribution of BED.

**Table 1:** Characteristics of HCC patients receiving SBRT from the NCDB (2004‐2013)

Variables (n)	Total cohort (n = 456)	Division by BED (Gy)			
		<75 (n = 141)	≥75 to ≤100 (n = 126)	>100 (n = 133)	*P*‐value
Age (y), median (range)	63 (57‐74)	64 (27‐89)	61 (48‐85)	66 (36‐90)	0.880
≥65 y	209 (45.3%)	65 (46%)	50 (40%)	59 (44%)	0.556
Gender Male	340 (73.7%)	103 (73%)	95 (75%)	94 (71%)	0.694
Ethnicity					
Caucasian	372 (80.7%)	117 (83%)	102 (81%)	108 (81%)	0.927
African American	51 (11.1%)	13 (9%)	13 (10%)	16 (12%)	
Others	38 (8.2%)	11 (8%)	11 (9%)	9 (7%)	
Insurance					
Not insured	16 (3.5%)	3 (2%)	6 (5%)	3 (2%)	0.663
Private	145 (31.5%)	43 (30%)	39 (31%)	45 (34%)	
Government	298 (64.6%)	94 (67%)	81 (64%)	85 (64%)	
Unknown	2 (0.4%)	1 (0.7%)	0 (0%)	0 (0%)	
Charlson‐Deyo Score					
0	281 (61.0%)	89 (63%)	68 (54%)	82 (62%)	0.135
1	91 (19.7%)	28 (20%)	34 (27%)	20 (15%)	
≥2	89 (19.3%)	24 (17%)	24 (19%)	31 (23%)	
AFP level					
Normal	130 (28.2%)	39 (28%)	37 (29%)	47 (35%)	0.626
Elevated	244 (52.9%)	79 (56%)	65 (52%)	64 (48%)	
Unknown	87 (18.9%)	23 (16%)	24 (19%)	22 (17%)	
Stage					
Stage I	257 (55.7%)	64 (45%)	74 (59%)	84 (63%)	0.002
Stage II	134 (29.1%)	44 (31%)	39 (31%)	37 (28%)	
Stage III	70 (15.2%)	33 (23%)	13 (10%)	12 (9%)	
Median tumor size (cm)	3.2 cm (0.6‐17.0%)	4.5 cm (1.1‐17.0)	3.0 cm (1.0‐10.8)	2.7 cm (1.0‐14.0)	<0.001
Chemotherapy					
None	303 (66.4%)	110 (78%)	64 (51%)	89 (67%)	<0.001
Yes	147 (32.2%)	29 (20%)	62 (48%)	39 (29%)	
Unknown	6 (1.3%)	2 (1%)	0 (0%)	5 (4%)	
Treating facility					
Academic	337 (73.9%)	104 (74%)	102 (81%)	100 (75%)	0.385
CCC	88 (19.3%)	25 (18%)	17 (14%)	27 (20%)	
Other	31 (6.8%)	12 (9%)	7 (6%)	6 (5%)	
Facility case volume					
>20	187 (41.0%)	47 (33.3%)	69 (54.8%)	61 (45.9%)	<0.001
6‐20	87 (19.1%)	46 (32.6%)	19 (15.1%)	19 (14.3%)	
1‐5	182 (29.9%)	48 (34.0%)	38 (30.2%)	53 (39.8%)	

The majority of the treatments in this US‐based cohort were delivered with 5‐fraction regimens (55% 5‐fraction, 39.5% 3‐fraction, and 3.9% 4‐fraction). The median BED was 100 Gy (22.5‐208.0), which corresponded to the most common dose fractionation (50 Gy in 5 fractions) in 23.0%. Other common schemes included 40 Gy in five fractions (BED of 72 Gy) in 15.3%, 48 Gy in three fractions (BED of 124 Gy) in 11.5%, 45 Gy in three fractions (BED of 112.5 Gy) in 6.8%, and 54 Gy in three fractions (BED of 151 Gy) in 4.3%. In comparison to commonly used regimens, patients who received 48 Gy in three fractions had a shorter time interval to SBRT, treatment at high‐volume academic facilities, received less chemotherapy, and had better outcomes than patients receiving other common regimens (Table 2). Patients treated with three fraction regimens tended to receive higher BED, receive treatment at nonacademic facility, and have a shorter time from diagnosis to SBRT. Various factors contributed to the selection of SBRT doses including stage, tumor size, facility case volume, and the use of chemotherapy.

**Table 2:** Comparisons between common fractionation schedules

Fractionation (BED in Gy)	n	BED (Gy)	Median age (y)	Stage 1/2/3	AFP elevated	Size median (cm)	Size groups ≤2/2‐4/>4 (cm)	Facility volume Low/mod/high	Academic facility	Charlson 0/1/2	Received Chemo	Time to SBRT <2/2‐4/>4 (m)	2 y OS
50 in 5 (100)	92	100	60.2	60/27/8	50%	2.8	26/56/18	26/10/64	89%	53/27/20	61%	13/39/48	43.4%
40 in 5 (72)	61	72	63.7	56/28/16	49%	3.1	22/38/40	27/25/48	87%	59/23/18	15%	41/38/21	44.8%
48 in 3 (125)	46	125	63.2	76/17/7	46%	2.4	37/50/13	15/2/83	94%	57/20/24	2%	50/30/20	79.0%
45 in 3 (113)	27	113	65.0	51/37/11	52%	3.5	24/44/32	59/15/26	63%	74/7/19	44%	26/44/30	52.4%
54 in 3 (151)	17	151	60.2	77/24/0	47%	2.1	50/38/12	47/0/53	77%	35/18/47	65%	24/47/29	70.1%
*P*‐value		<0.001	0.411	0.116	0.309	0.006	0.014	<0.001	0.003	0.114	<0.001	<0.001	<0.001
3‐fractions	158	114	62.3	58/28/14	53%	3.27	25/43/33	30/18/51	70%	65/14/22	26%	35/41/24	50.8%
5‐fractions	220	98.9	62.9	54/32/14	51%	3.10	21/44/36	31/20/48	85%	57/24/19	37%	27/36/36	42.3%
*P*‐value		<0.001	0.370	0.624	0.174	0.044	0.678	0.784	0.001	0.064	0.013	0.032	0.083

**Figure 2:**
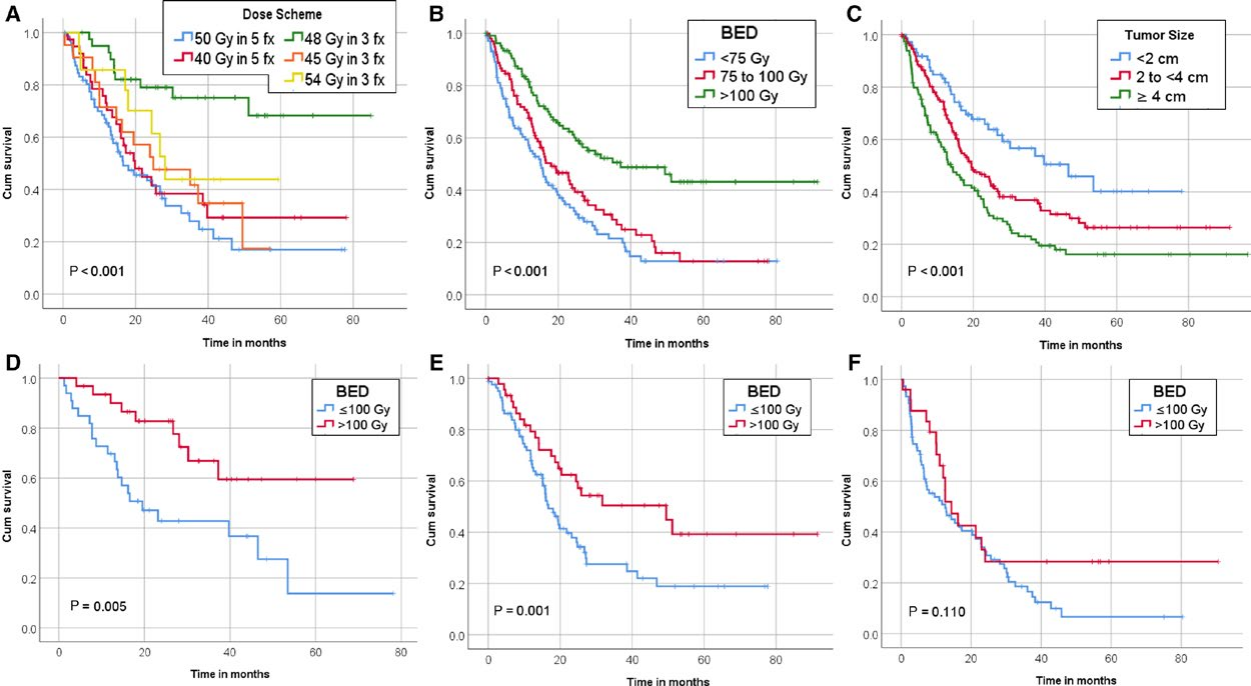
Kaplan Meier curves for overall survival: A, Commonly used regimens; B, BED; C, Size; D, Tumors <2 cm; E, Tumors 2 cm to <4 cm; F, Tumors ≥4 cm

After a median follow‐up was 16 months, a total of 217 (60.9%) patients had died. Patients alive at last encounter had a median follow‐up of 31 months compared to the 16 months for patients who died. The median OS after SBRT for the entire cohort was 20.3 months. Long‐term survival was observed with 75% of those surviving longer than 3 years alive at last follow‐up (range 36.2 to 91.4 months, median 54.25 months). Increasing BED was associated with improved survival. The median and 1‐year OS were 15.3 months and 56.6% for BED ≤ 75, 18.3 months and 67.5% for BED > 75 and ≤100, and 37.2 months and 81.4% for BED > 100, *P* < 0.001. Increasing tumor size also correlated with worse outcome. The median and 1‐year OS were 46.5 months and 81.3% for size ≤2 cm, 19.5 months and 70.6 for size >2 cm and ≤4 cm, and 15.1 months and 58.5% for size >4 cm, *P* < 0.001 (Figure 2). BED greater than 100 Gy did not benefit all tumor sizes equally. For tumors greater than 4 cm, there was no improvement with BED greater than 100 Gy, but for tumors ≤4 cm, there was an improvement in overall survival (Figure 2).

**Figure 3:**
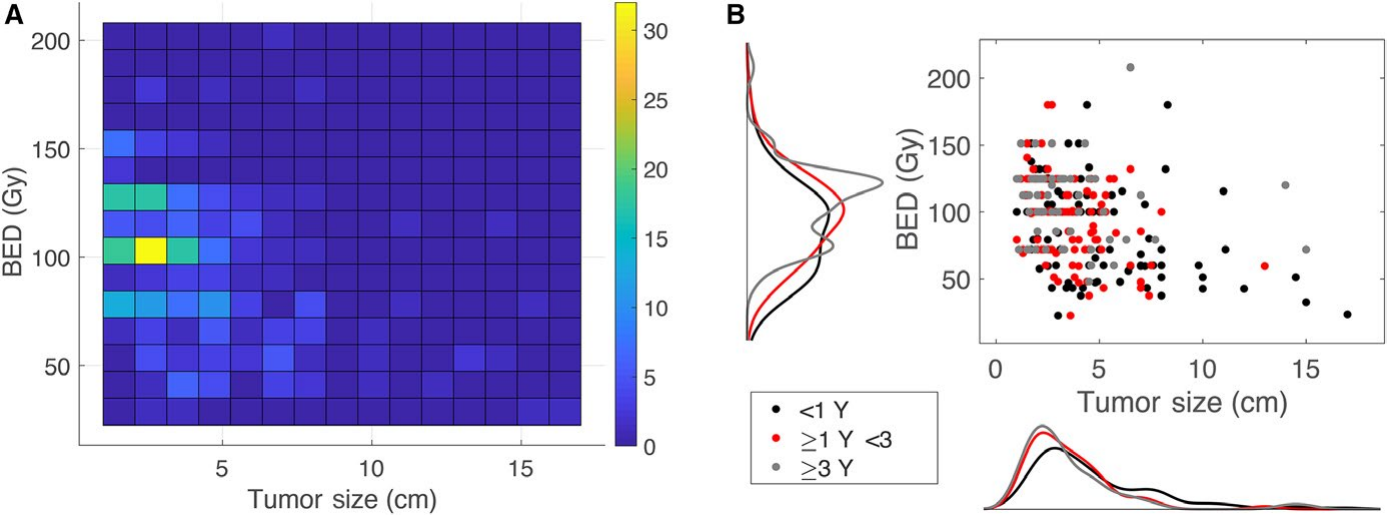
Relationships between BED, tumor size, and survival. A, Heat map showing the frequency of BED and tumor size; B, Scatter plot and frequency curves of BED and tumor size divided into survival groups (<1 y, 1 to <3 y, and ≥3 y)

On multivariate analysis, low BED, elevated AFP, larger tumor size, increased interval from diagnosis to SBRT, and low facility SBRT volume were associated with worse survival (Table 3). There was an inverse relationship between the size of the tumor and the BED in relation to survival. For patients surviving less than 1 year, more than 1 year to 3 years, and more than 3 years, the median tumor size decreased from 3.75 cm to 3.0 cm to 2.7 cm (*P* < 0.001), while the median BED increased from 92.75 Gy to 100 Gy to 112.5 Gy (*P* < 0.001), respectively. Correlation between tumor size, BED, and survival is shown in Figure 3.

**Table 3:** Univariate and multivariate analysis for overall survival

Category	Univariate analysis			Multivariate analysis		
	Hazard ratio	95% Confidence interval	*P*‐value	Hazard ratio	95% Confidence interval	*P*‐value
Age continuous variable	1.009	0.997‐1.021	0.153			
Sex (male (ref) vs female)	0.998	0.740‐1.346	0.99			
Charlson‐Deyo Score						
0	(ref)	(ref)	(ref)			
1	1.086	0.752‐1.568	0.661			
2	1.144	0.736‐1.777	0.55			
AFP Normal (ref)	(ref)	(ref)	(ref)	(ref)	(ref)	(ref)
AFP Elevated	1.617	1.145‐2.284	0.006	1.585	1.078‐2.329	0.019
AFP unknown	1.996	1.335‐2.983	0.001	1.865	1.160‐2.999	0.010
Tumor size (cm) (continuous)	1.119	1.072‐1.169	<0.001	1.067	1.006‐1.131	0.032
Stage						
1	(ref)	(ref)	(ref)	(ref)	(ref)	(ref)
2	1.326	0.974‐1.805	0.073	1.326	0.974‐1.805	0.073
3	1.962	1.384‐2.780	<0.001	1.962	1.384‐2.780	<0.001
Treating facility						
Academic	(ref)	(ref)	(ref)			
CCC	1.188	0.644‐2.191	0.581	NS	NS	NS
Other	1.942	1.013‐3.725	0.046			
Facility case volume						
>20	(ref)	(ref)	(ref)	(ref)	(ref)	(ref)
6‐20	1.814	1.341‐2.454	<0.001	1.324	0.862‐2.003	0.200
1‐5	2.101	1.453‐3.038	<0.001	1.687	1.165‐2.442	0.006
Months from diagnosis to SBRT						
≤2 mo	(ref)	(ref)	(ref)	(ref)	(ref)	(ref)
>2 mo to ≤4 mo	1.468	1.055‐2.043	0.023	1.456	1.004‐2.113	0.048
>4 mo	1.422	0.955‐2.031	0.053	1.586	1.069‐2.353	0.022
BED						
>100 Gy	(ref)	(ref)	(ref)	(ref)	(ref)	(ref)
≥75 Gy and ≤100 Gy	1.986	1.371‐2.875	<0.001	1.698	1.158‐2.490	0.007
<75 Gy	2.537	1.767‐3.643	<0.001	2.192	1.485‐3.235	<0.001

## 4 DISCUSSION

Due to improvements in technology and an enriched understanding of the liver's tolerance of radiation, stereotactic radiation body radiation therapy has emerged as a promising treatment for unresectable HCC. There is growing acceptance and application of this technique for managing HCC as shown by the dramatic rise in its use over the study period. Despite the growing availability of liver SBRT, there remains little consensus about the best treatment regimen.^18^ This study confirms the wide variation of regimens used in clinical practice and identifies 50 Gy in five fractions as the most common scheme in this cohort of patients treated in the United States, which is also the recommended dose on the currently enrolling RTOG 1112 protocol, if liver constraints can be achieved. The variation in treatment regimens reflects the multiple factors that are considered in determining the dose including the size and location of the lesion, the radiation tolerance of nearby organs, liver health, the amount of liver spared from significant radiation doses, institutional preference, and organ motion managment.^5^, ^18^, ^19^

Given the variation in doses and fractionation schemes used, all of the doses were converted to Biological Effective Dose (BED) in order to accommodate direct comparisons between different regimens^17^ and evaluate the impact of dose and outcome. Correlation between dose, tumor size, and overall survival emerged, which is partially explained by the aforementioned factors as patients with the most favorable factors likely were given higher BED prescriptions. In this cohort, those receiving high BED treatments tended to have smaller tumors, normal AFP, and earlier tumor stage than those who received low BED treatments. Other studies have also reported an inverse relationship between the size of the tumor and the prescription dose for SBRT.^6^, ^20^, ^21^ The specific dose‐fractionation scheme may be less important than safely treating to the appropriate BED for each patient's clinical situation.

It has been over 20 years, since the first clinical experience with SBRT for HCC showed efficacy.^22^ This was followed by multiple retrospective and prospective reports further supporting and validating SBRT as a promising option for HCC.^6^, ^7^, ^8^, ^9^, ^10^, ^11^, ^12^, ^15^ Table 4 summarizes select studies. Among the published studies, overall survival varies considerably based on the cohort composition. The current study is the largest evaluating overall survival and practice patterns from a diverse group. Based on BED, it reports similar survival outcomes compared to other published reports (55% 1‐year OS for Bujold et al with median BED of 54 Gy vs 56.6% 1‐year OS for patients with BED <75 Gy from current study and 61%‐67% 3‐year OS reported by Kang et al and Lasley et al with median BED of 124‐165 Gy vs 50.6% 3‐yr survival for BED >100 Gy in the current study). Although some variation is expected given the increased cohort heterogeneity and decreased selectivity compared to prospective trials, it appears the outcomes seen with SBRT in practice are reasonably comparable to previous reports.

**Table 4:** Selected studies of stereotactic body radiation therapy for HCC

Study	Median follow‐up (m)	# of patients	Median dose/BED	Median tumor size	Local control	Overall survival	Toxicity	Comments
Bujold^6^	31 (2‐36)	102	36 Gy in 6 fx (24‐54)	117 cc/7.2 cm	1‐y 97%	Median 17 m	30% Grade ≥3	55% with PVT
			BED: 57.6 Gy (33.6‐102.6)			1‐y 55%		61% with multiple lesions
Lasley^7^	CPA: 33	59	CPA: 48 Gy (36‐48 Gy/3 fx)	34 cc	1 year	Median/3‐year	Grade≥3	20% with PVT
	CPB: 46	CPA: 39	BED: 124 Gy (79.2‐124)		CPA: 91%	CPA: 44.8 m/61%	CPA: 11%	
		CPB: 21			CPB: 82%	CPB: 17.0 m/26%	CPB: 38%	
			CPB: 40 Gy/5 fx					
			BED: 72 Gy					
Kang^8^	17 m (6‐38)	47	57 Gy in 3 fx (42‐60 Gy in 3 fx)	14.9 cc/2.9 cm	2 y 95%	2‐y 69%	6.4% Grade ≥3	100% had prior TACE procedure
			BED: 165.3 Gy (100.8‐180)					
Takeda^9^	41	90	40 Gy in 5 fx (35‐40 in 5 fx)	2.3 cm	3 y 96.3%	Median 54.7 m	Total Gr 3 toxicity of 16.7%,	42% recurrences after other prior therapies
			BED: 59.5‐72 Gy			3‐y 66.7%	No Gr 4‐5	
Scorsetti^10^	8	43	<3 cm: 48‐75 Gy in 3 fx	4.8 cm	1‐y	Median/1‐y	16% Grade ≥3	47% CPB, 20% PVT
			BED: 124.8‐262.5 Gy		All patients: 86%	18 mo/78%		
					BED>100: 100%			
			3‐6 cm: 36‐60 Gy in 6 fx		BED<100: 52%			
			BED: 57.6‐120 Gy					
Bibault^11^	10	75	45 Gy in 3 fx (24‐45 Gy in 3 fx)	3.7 cm	1 y 89%	1‐y 78.5%	Gr 3 in 8%	51% prior other tx, CPB 12%,
			BED: 112.5 Gy (43.4‐112.5 Gy)				Gr 4 in 1%	
Sanuki^12^	24	185	40 Gy in 5 for CPA, BED 72 Gy	7.2 cc	3‐y 91.6%	3‐y 72.1%	1.5% Grade 3	~70% had prior liver treatment, 15% CPB, 84% stage I
			35 Gy in 5 for CPB, BED 59.5 Gy	8.9 cc	3 y 90.7%	3‐y 66.0%	8.3% Grade 3, grade 5 in 7% of CPB	
Su^15^	21	132	42‐46 Gy in 3‐5 fractions	3 cm	1 y 90%	3‐y 73.5%	Gr ≥3 of 8.3%	CPB in 14%,
			BED: 78‐115 Gy					
Current Study	16	362	23%‐50 Gy in 5, BED 100	3.2 cm	NA	Median 20 m	NA	12% stage 3, >50% with elevated AFP
			15.3%‐40 Gy in 5, BED 72			1‐y 69.7%		
			11.5%‐48 Gy in 3, BED 124			3‐y 36%		

The current study suggests that elevated AFP, larger tumor size, stage 3 disease, low facility case volume, increased time interval from diagnosis to receiving SBRT, and low BED are associated with poor survival for unresectable HCC patients treated with SBRT. While several of these factors have been identified as prognostic factors in other studies (elevated AFP,^9^, ^23^ tumor size,^11^, ^21^, ^23^, ^24^ SBRT dose,^8^, ^10^, ^11^, ^25^ advanced stage^6^, ^26^), low facility case volume and increased time interval from diagnosis to receiving SBRT are new and may warrant further study. Since liver SBRT is a relatively new technique, facility volume and provider experience may influence results similar to that of surgical outcomes for complicated oncologic cancers^27^, ^28^ or when intensity‐modulated radiation therapy was first introduced for head and neck cancer treatment.^29^ Each of these situations requires mastering complicated procedures, understanding complex anatomy, and managing multiple prognostic variables similar to that needed for liver SBRT. It could also reflect better practices for preventing liver toxicity or advanced technology in place at busier centers that could improve SBRT efficacy and safety such as advanced image guidance, established respiratory motion strategies, and MRI simulation. To this point, the Princess Margaret experience showed improved outcomes in patients treated on their second trial, which they attributed to better patient selection, diagnostic imaging, target identification, and radiation planning/delivery.^6^ Additionally, the University of Michigan group reported no local failures using fiducials to enhance image guidance compared to 10% failure rate without fiducials, which further highlights the importance of image guidance and the technical components of SBRT treatment. These factors are developed and enhanced with expertise and experience from larger case volumes. A longer time interval from diagnosis to SBRT may be associated with a worse outcome due to tumor growth and invasion leading to more advanced tumors at the time of SBRT, worsening liver function over time, or could reflect failed initial treatments, which could make SBRT more complicated and less efficacious. In the current study, patients with prior therapy had a longer time from diagnosis to SBRT than those without prior therapy.

Multiple groups have evaluated the impact of SBRT dose on outcomes with several reporting improved local control with increased dose.^8^, ^10^, ^11^, ^25^ Scorsetti et al^10^ reported 100% local control for tumors that received a BED dose of >100 Gy vs 52% for those with <100 Gy BED. Likewise Kang et al^8^ reported improved 2‐year local control (100% vs 87%) and progression‐free survival (52.5% vs 17.3%) for BED ≥151 Gy vs <151 Gy. Additionally, Jang et al^25^ reported not only improved local control, but also improved overall survival for patients receiving a BED ≥151 Gy compared to lesser dose regimens. To the contrary, several groups found no relationship between dose and outcome. In series of small tumors with the majority receiving high‐dose treatments, neither Su et al nor Wahl et al reported a difference in outcome‐related SBRT dose.^13^, ^15^ They speculated that their delivered dose even in the lower dose patients was sufficiently high to achieve local control.^13^ In the Princess Margaret cohort of large advanced tumors, no difference in local control was observed with increased SBRT dose, but most tumors were treated with relatively low BED due to the risk of liver failure (median BED was 57.6 Gy).^6^ The current study shows improved outcomes with increasing BED dose, particularly in those who received a >100 Gy BED, but the benefit of high dose was less significant in patients with larger tumors. This could be due to the inability to achieve a high dose in these larger tumors while respecting normal liver constraints, or possibly, from liver related toxicity in those with large tumors inappropriately treated with high doses. There are many challenges in evaluating the role of dose from previous reports including: homogeneous patient populations, only minor differences in evaluated SBRT dose regimens, and small sample sizes. The current study adds to the discussion about dose, because it includes a large patient population with a broad range of SBRT doses and practice patterns. Patient selection likely contributed significantly to the overall survival benefit that was observed. The positive impact of dose on outcomes for unresectable HCC is also supported by several older studies with more traditional extended fractionation.^30^, ^31^, ^32^ Likely, the true value of escalated dose depends on multiple factors, particularly whether it can be done safely.^5^, ^33^ Efforts to use modern radiation techniques or even newer therapies like proton beam radiation to stay within organ constraints and apply recommendations from predictive models to individually access the risk of liver toxicity with different doses are warranted.^5^, ^33^

Several significant limitations of this study relate to its retrospective nature and the available information in the database. The NCDB database does not include information about tumor location, tumor number, Child Pugh status, treatment related toxicity, local control, or cause of death, which are key factors related to liver SBRT. Of these the most critical is the lack of information related to liver function, as this is a key determinant in deciding what dose to use and if SBRT is a safe option for the patient. Using too high of a BED scheme in a patient with poor liver function can result is severe toxicity and even death.^34^ Additionally, some elements of the radiation treatment are not reported in the database such as the isodose coverage, maximal/minimal dose, margin expansion, or dose to the organs‐at‐risk most importantly the liver. As such these results should be considered as exploratory in nature and not evidence to systematically apply higher BED regimens in all cases. Despite these limitations, this study provides insight into current practice patterns and suggests some relationships that may be useful for improving outcomes, and understanding the role of SBRT for unresectable HCC. The development of techniques and strategies to safely deliver high BED regimens may further enhance treatment, while limiting the risk of potential acute side effects and late toxicities. Further study may better elucidate a specific dose threshold for excellent tumor control and low toxicity.

In summary, SBRT is an effective option for unresectable HCC and is growing in its use and application in clinical practice. Multiple fractionation schemas are used and several factors including tumor size, stage, and facility volume likely play a role in the selection process. Higher BED treatments may help improve outcomes in properly selected patients, but care must be taken to ensure it can be done without significantly increasing the risk of toxicity. Additional study on factors affecting SBRT outcome and the role of SBRT in treating HCC is warranted.

## CONFLICT OF INTEREST

No financial support or conflict of interest to disclose in relationship to this study.
